# Method for the biomechanical analysis of aqueous veins and perilimbal sclera by three-dimensional photoacoustic imaging and strain field calculation

**DOI:** 10.1038/s41598-021-01458-1

**Published:** 2021-11-11

**Authors:** Linyu Ni, John Riesterer, Huaizhou Wang, Layla Berry, Kara Blackburn, Jonathan Chuang, Wonsuk Kim, Guan Xu, Sayoko E. Moroi, Alan Argento

**Affiliations:** 1 Department of Biomedical Engineering, University of Michigan, Ann Arbor, MI 48109 USA; 2 Department of Mechanical Engineering, University of Michigan-Dearborn, 4901 Evergreen Road, Dearborn, MI 48128 USA; 3 Department of Ophthalmology and Visual Sciences, Kellogg Eye Center, University of Michigan, Ann Arbor, MI 48105 USA; 4 Currently at Beijing Tongren Eye Center, Beijing Tongren Hospital, Capital Medical University, Beijing, China; 5 Department of Ophthalmology and Visual Sciences, Havener Eye Institute, The Ohio State University Wexner Medical Center, Columbus, OH 43210 USA

**Keywords:** Biomedical engineering, Optical techniques, Glaucoma, Mechanical engineering

## Abstract

A method motivated by the eye’s aqueous veins is described for the imaging and strain calculation within soft biological tissues. A challenge to the investigation of the biomechanics of the aqueous vein—perilimbal sclera tissue complex is resolution of tissue deformations as a function of intraocular pressure and the subsequent calculation of strain (a normalized measure of deformation). The method involves perfusion of the eye with a contrast agent during conduction of non-invasive, optical resolution photoacoustic microscopy. This imaging technique permits three-dimensional displacement measurements of tracked points on the inner walls of the veins which are used in a finite element model to determine the corresponding strains. The methods are validated against two standard strain measurement methods. Representative porcine globe perfusion experiments are presented that demonstrate the power of the method to determine complex strain fields in the veins dependent on intraocular pressure as well as vein anatomy. In these cases, veins are observed to move radially outward during increases in intraocular pressure and to possess significant spatial strain variation, possibly influenced by their branching patterns. To the authors’ knowledge, these are the only such quantitative, data driven, calculations of the aqueous vein strains available in the open literature.

## Introduction

Despite knowledge of clinical risk factors and effective treatments to lower intraocular pressure (IOP)^1^, glaucoma is a major cause of blindness worldwide^2^. Based on the classic study on enucleated human eyes, the major site of outflow resistance is the trabecular meshwork (TM), which is also called the conventional outflow pathway^3^. The remaining resistance distal to the TM includes Schlemm’s canal, the collector channels, and aqueous veins within the perilimbal sclera^4^. In human eyes, there is regional variation of aqueous humor outflow through the TM into Schlemm’s canal^5^ and the collector channels^6^.

The role of perilimbal sclera tissue biomechanics on the aqueous veins is relevant to the understanding of IOP regulation beyond the TM. For the newer glaucoma surgeries that target the TM, it is assumed that the IOP will decrease because of improved egress of the aqueous humor beyond the TM, into Schlemm’s canal and into the distal aqueous veins. Given the variable post-surgical outcomes^7^ to lower IOP with such glaucoma surgeries, it is clear that there exists a gap in knowledge concerning the distal aqueous outflow and the biomechanical interactions within the perilimbal scleral tissue. Advancing knowledge of the aqueous veins and perilimbal sclera biomechanics will begin to fill this knowledge gap. In a prior study^8^, we performed perfusion experiments to determine outflow facility in enucleated porcine eyes followed by mechanical stress–strain tests of sclera using tissues from these same eyes. The results from these experiments showed that increased mechanical stiffness of the perilimbal sclera significantly correlated with increased steady-state outflow pressure.

A challenge to the investigation of the biomechanics of the aqueous vein—perilimbal sclera tissue complex is resolution of tissue deformations as a function of IOP and the subsequent calculation of strain, particularly in three dimensions. Conventional optical microscopy has limited tissue penetration and therefore cannot resolve the vasculature within the sclera. Optical coherence tomography (OCT) and OCT angiography have the capability to visualize the vasculature in the anterior segment of the eye, including aqueous veins^9–12^. In OCT images of perfused research eyes, the vasculature appears as void regions within the sclera^10^. Following empirical adjustments of the image contrast in the A-scans and complicated post-processing methods, the vessel contours of the aqueous veins may be extracted^11^. Significant segmentation artifacts have been observed in OCT, which may introduce errors if the images are subsequently used to compute strain based on spatial feature tracking^13^. OCT angiography possesses unique advantage in resolving vasculature in vivo, although active flow of optical scatterers, such as blood cells, are required for detection^14^. The aqueous veins that are proximal to the collector channels from Schlemm’s canal are relatively acellular as these veins are conduits for the aqueous humor from the anterior chamber and the TM or conventional outflow pathway. The limited red blood cells in aqueous veins are not sufficient to provide scattering contrast for OCT and clear definition of the aqueous veins boundaries for accurate strain analysis. In addition, differentiating among episcleral, intrascleral, and aqueous veins in vivo requires empirical thresholding^9^ and, therefore, involves uncertainty when aqueous veins are of particular interest. Although these works resolved the anatomies of the aqueous veins, deformations were not measured and strains were not calculated.

Experimental determination of strains on the outer surface of sclera has been conducted in a number of studies. For example, camera imaging of tracking targets applied to the posterior surface of whole porcine globes was used^15^ to determine two-dimensional (2-D) displacements for computation of the biaxial strains. Three-dimensional (3-D) surface strains were calculated after measurements of surface displacements of a posterior, hemispherical portion of human sclera by laser speckle interferometry^16^. The results revealed that tensile surface strain of the parapapillary sclera was statistically significantly greater than the mid-peripheral sclera. The paper only gives measurements of surface strain. Calculation of strain within soft tissue demands an imaging modality that can penetrate the tissue, which is more difficult. An earlier study^17^ used an ultrasound elasticity microscope in conjunction with finite element methods to determine strains within porcine corneal tissue. In the method, the globe’s sclera is entirely embedded in stiff gelatin and the cornea is submerged in water above the gelatin. The purpose of the study is refractive surgery and results were given for only the cornea. Another work on cornea used displacements measured by ultrasound speckle tracking to determine the strains through the tissue’s thickness by direct numerical calculation^18^. The results show in-plane stretching of cornea with through the thickness compression, as also found in the present manuscript for sclera. However, ultrasound imaging has limited spatial resolution and cannot distinguish the deformation of vasculatures from that of the sclera tissue. The biomechanics of the connected cornea-limbus-sclera tissues of the porcine eye was studied in^19^. Variations in elastic properties as a function of IOP were inferred from measurements of the speed of propagating Lamb waves induced in the tissue. Strains were not measured, but finite element simulations of the tissues provide validation of the measured trends. The strains within the tissues of human lamina cribrosa and adjacent sclera were recently studied in^20^. Whole globes were resected into hemispherical sclera segments that were fixed to a support along the cut edge. Imaging was conducted by laser-scanning microscopy and digital volume correlation to determine displacement fields from which strains were directly calculated from the strain–displacement equations. As in the present manuscript, the method is able to resolve tissue displacements within the sclera with sufficient accuracy for strain calculations.

Imaging the aqueous vein—perilimbal sclera tissues for biomechanical study demands penetration into the sclera and resolution of the micron-scale vasculature. To the authors’ knowledge, no studies are available on experimental determination of deformations and strains in these tissues in a whole globe.

Photoacoustic imaging is a non-invasive and non-ionizing imaging modality that has high sensitivity to the optical absorption contrasts in biological tissue^21,22^. During photoacoustic imaging, pulsed illumination with a temporal width of nanoseconds generates acoustic waves that are captured by ultrasound sensors. Since the transmission of acoustic waves in tissue results in significantly less scattering and attenuation than transmitted optical signals, photoacoustic imaging has the advantage of deeper penetration compared to conventional optical microscopy^22^. The method has been implemented in ophthalmologic imaging^23,24^ with optical energy as low as 1% of the safety limit established by American National Standard Institute^25^.

In this paper, a new method is described that uses optical resolution photoacoustic microscopy to capture 3-D displacement fields within soft tissue along with finite element analysis (FEA) to calculate the corresponding strains based on the measured data^26^. The method is particularly relevant to the eye’s aqueous veins. In the method, enucleated eyes are perfused with a contrast agent that enters the aqueous veins and stains their inner walls, thereby allowing points on the vein walls to be imaged and tracked by photoacoustic microscopy as intraocular pressure is varied. A deforming finite element mesh is created from the movement of the tracked points that is used to calculate the resulting strains in the vein tissues. Results are given in which the method is validated by conduction of error analysis and comparison of the calculated strain fields to other measurement techniques. Whole porcine globe perfusion experiments are conducted that demonstrate the utility of the method to reveal the biomechanical response of eye’s aqueous veins due to outflow and changes in IOP.

## Materials and methods

### Eye preparation

Porcine eyes were obtained from two certified slaughterhouses: Milligan’s Northwest Meat Market (Jackson, MI) and Scholl’s Slaughterhouse (Blissfield, MI). Eyes were transported in closed plastic bags in a cooler with ice, then stored at 4 °C with all fat intact to prevent desiccation of tissues. Preparation and handling of samples followed the procedure described previously^27^. All fat, extraocular muscles and peribulbar tissues were removed. The optic nerve was trimmed flush to the scleral surface. Eyes were hydrated during preparation by periodically moistening with balanced salt solution (BSS). Before testing, each eye was gradually warmed to 37 °C for about 15 min by inserting it in a warmed hydration tube containing gauze moistened with BSS. This maintains humidity in the vials at about 95%, as monitored by a humidity probe inserted in the vial^27^. A laser thermometer was used before testing to verify that the eye was at physiological temperature.

**Figure 1:**
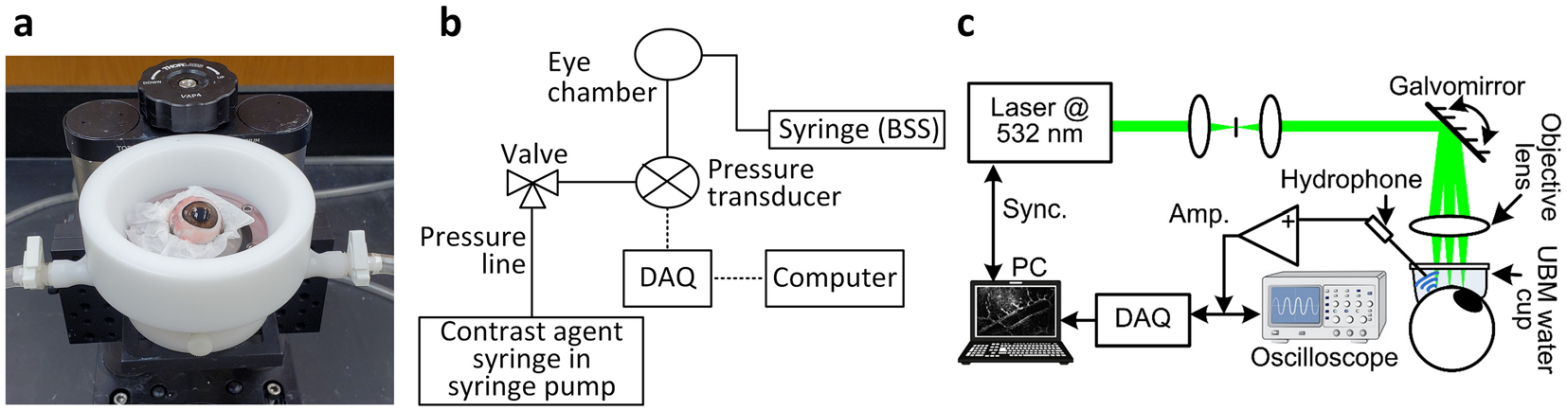
Experiment setup. (**a**) Globe holder and hydration system, (**b**) perfusion system, (**c**) photoacoustic imaging system.

After preparation, tests were conducted with the eye in a custom built apparatus consisting of a water-jacketed globe holder and plastic spacer that slots on the rim of the holder (Fig. 1a). A water heating and circulation system circulates water at 37 °C through a closed chamber in the water-jacketed globe holder that encircles the eye, as shown. This serves the function of warming the globe without allowing water to directly contact it. Tissue wipes moistened with BSS were placed under the eye to maintain hydration, raise it slightly off the bottom surface and permit minor adjustments to eye position for imaging. No glue or fixation was used since these produce stress and strain focally near the attachment points. This support system allows the eye to freely expand during pressure changes.

### Preparation and use of contrast agents

To facilitate photoacoustic imaging, a contrast agent that creates contrast and marks features in the tissue is usually needed. In the case of pigmented sclera, which sometimes occurs in porcine eyes, a contrast agent is not needed because the BSS-filled aqueous veins appear clear against the pigmented sclera. In the present case, since the aqueous veins are void of circulation of blood cells, a contrast agent is needed for the photoacoustic imaging in this study.

It is necessary that the imaging agent perfuse the venous system without blocking the vessels, and with minimal diffusion from the aqueous veins and into the scleral tissue surrounding the veins, which would obscure photoacoustic imaging of the veins. Imaging trials were conducted using red tattoo ink (“Dragon’s Breath,” Kuro Sumi Corp., Fort Mill, SC), indocyanine green, trypan blue and fluorescent nanoparticles. Trypan blue passed into the veins but tended to diffuse through the veins and stain the surrounding sclera. Fluorescent nanoparticles were found to enter the veins but many particles tended to pass out of the veins and lodge in the sclera adjacent to the veins. The tattoo ink (diluted from stock with BSS to 5%) and indocyanine green (mixed at its stock concentration of 0.25%) were found to readily enter the veins after preconditioning with BSS and identify aqueous veins in contrast against nonpigmented, white sclera with only slight leakage into the surrounding sclera. In some cases, it was necessary to conduct a partial trabeculotomy in order for the tattoo ink to pass into the veins but this was not necessary for the indocyanine green. Green light at 532 nm targeting the red color was used for the red tattoo ink and achieved strong PA signals. Since only structural information is needed and no other optical contrast is included in this study, the specific optical absorption profile of the red ink was not investigated.

### Perfusion set-up and process

The arrangement of the perfusion system (Fig. 1b) included 3/32 inch flexible tubing, a pressure sensor (Argon DTXPlus Model DT-XX, Argon Critical Care Systems Singapore Pte. Ltd, Singapore), two 21 gauge needles, two 10 cc syringes, a syringe pump and a data acquisition system. One syringe was filled with BSS and the other with a contrast agent. Air bubbles were removed from the system by slowly drawing the fluids in and out of the tubing and sensor until all bubbles clear. Next, the pressure sensor was zeroed. The needle of the BSS line was inserted through the cornea and into the posterior chamber. The contrast agent needle was inserted into the anterior chamber. For the presented perfusion examples, the needle was guided carefully to the drainage angle and gently, yet firmly used to cut through an approximately 45° arc segment of the trabecular meshwork to perform a trabeculotomy. The trabeculotomy decreases resistance and allows the tattoo ink contrast agent to access the aqueous veins more readily. The imaging agent syringe was then placed in the syringe pump, set to off position.

The BSS line was then used to precondition the eye via a pressurization/relaxation process by which the enucleated eye’s tissues are returned to near physiological biomechanical condition^8^. Here, this process also serves to prime the aqueous veins for subsequent perfusion with the contrast agent. Once the preconditioning process was complete, the BSS syringe was closed, and the imaging system was focused and prepared to collect data.

The IOP was then increased by perfusion with the contrast agent at a constant flow rate of 9 µl/min. Depending on the particular eye, the agent was observed by white light imaging to enter the aqueous veins at IOP less than roughly 22–28 mmHg. When a clear pattern of veins appeared, the flow rate was reduced and white light and photoacoustic images were obtained as the IOP either increased or decreased. During this early perfusion period, images were taken at a near-zero strain state when IOP was between 4–6 mmHg. Strain at all the other IOP levels were calculated relative to the reference image obtained at near-zero strain.

### Photoacoustic imaging process

All the photoacoustic microscopy images in this study were captured by a system with lateral and axial resolution of 4.1 µm and 37 µm, respectively^28^. The configuration is briefly illustrated in Fig. 1c. In the system, illumination at 532 nm wavelength is generated by a pulsed diode laser (L4, Elforlight, United Kingdom) at a pulse duration of 5 ns and repetition rate of 5 kHz. The light beam is spatially filtered and collimated in the axial direction into a diameter of 3 mm, and scanned in two dimensions laterally by a galvomirror. A telecentric lens (focal length 36 mm) focuses the scanning beam at the plane of imaging. An immersion technique is used to propagate the photoacoustic signals from the eye and through liquid. This immersion consists of a cellophane reservoir with a clear and smooth contact to the ocular surface. A needle hydrophone (50 MHz central frequency with 50% bandwidth at −3 dB range, customized by The Ultrasound Institute at The University of Southern California) is placed in the liquid and captures the signals. The signals are amplified by 57 dB, displayed in real time by an oscilloscope, digitized by a DAQ card and stored in the personal computer (PC).

In this study, the acquisition of each A-line signal encoding optical contrasts along the axial dimension of the scanning light beam, limited by the laser repetition rate of 5 kHz, takes approximately 20 µs. Lateral 2-D spatial scanning of the light beam, in addition to the signal arrival time in A-line, forms a 3-D map of the optical contrast within the region of interest. The scanning step size was 4–10 µm, depending on the area of the region of interest. The images shown later were generated by projecting the maximum peak-to-peak values in the A-line signals. During the mechanical test for each sample, a series of photoacoustic images were taken along with the variation of IOP.

### Feature tracking and finite element mesh generation

For quantifying the deformations within the tissue, the displacements of the spatial features in the photoacoustic image series were first tracked automatically in 2-D or 3-D. The method used is first described for the 2-D case. Due to the laser fluctuation and system noise, points with low contrast may not be traced through the whole strain experiments. To ensure reliability, multiple contact thresholds were tested to identify pixels with at least 55% contrast over any of their neighboring ones in the arbitrary reference state (a near-zero strain state) of the image series for iterative tracking. A 21 × 21 pixel image patch area with the tracked pixel at the center was first selected. The center of a sliding window with the same dimension then scanned a 61 × 61 pixel area around the current location of the tracked pixel in the following frame. The location in the scanned area with the largest correlation coefficient was determined as the new location of the tracked feature^29^. For ensuring tracking accuracy, the procedure was empirically repeated with a patch size of 31 × 31 and 41 × 41 pixels. If the new locations derived with the three patch sizes fell in a narrow range of 2 pixels, the new location was determined as the averaged location rounded to the nearest pixel. Otherwise, the current pixel was disregarded and the process proceeded to the next pixel.

The rationale for the selection of window sizes is based on the necessity to have a window size smaller than the vessel and larger than the displacements per step. The diameters of the aqueous veins tracked in these eyes are on the order of 150–200 µm and the lateral displacement of the spatial features are on the order of 15–20 µm in each step. Therefore, scanning windows were used having lateral dimensions of at least 80, 120 and 160 µm (corresponding to the window sizes of 21 × 21, 31 × 31 and 41 × 41 mentioned above). This method produced displacements from at least 100 tracked pixels in a 100 × 100 µm^2^ region which are appropriate to realistically represent the deformed shapes of veins. The same method was used in the 3-D tracking algorithm, except the correlation coefficients were calculated using 21 × 21 × 71, 31 × 31 × 101 and 41 × 41 × 133 pixel sliding windows, the center of which scanned over a 61 × 61 × 101 voxel space around the tracked voxel. The axial dimensions of the windows were determined as approximately 3.3 times the lateral dimensions, since the axial resolution is lower than the lateral resolution.

Optical contrasts for tracking points were created by passage of the contrast agent into the aqueous veins which stain the veins thereby providing trackable points, as described above. Here, strains in the veins were calculated directly via the tracked displacements of stained points on the deforming veins. The average strain in a segment of sclera bordered by stained veins was also calculated using the displacements of points on the veins.

In any case, the points tracked through all the frames were used for finite element mesh generation. Aimed at element size uniformity, one point was retained within any surface area with a radius of approximately 30—300 µm, depending on the size of images, in the reference frame. The retained points were defined as the nodes in the finite element mesh. The element lists were generated using the coordinates of the nodes with a built-in function, delaunayTriangulation, in MATLAB.

### Strain calculation

To calculate strains, the set of generated elements at the near-zero strain state was imported into the finite element package LS-DYNA (version R9.0, Livermore Software Technology, Livermore, CA). This results in a mesh of constant-strain, solid elements and the strain engine of the program is used to calculate the 3-D strains of the elements. At each nodal point of the elements the average strains of connected elements are determined, and then the nodal strain values are interpolated within the elements to determine the spatial distributions of the strains. In the developed finite element model, the tracked displacement vectors between image frames were incorporated as prescribed nodal motions and the resulting strain fields computed at various intraocular pressures. We used a similar method to determine the strain fields in scleral collagen fibers in a recent study^26^. The presented strains were computed using the linear, true strain model in LS-DYNA.

In some results, the average strain (denoted by an overbar, as in $\overline{\upvarepsilon }$) of an imaged region is computed. This is defined as the mean of the element strains weighted by the initial, undeformed, area or volume of each 2-D or 3-D element, respectively.

## Results

### Three-dimensional strains in porcine aqueous veins (Example 1)

**Figure 2:**
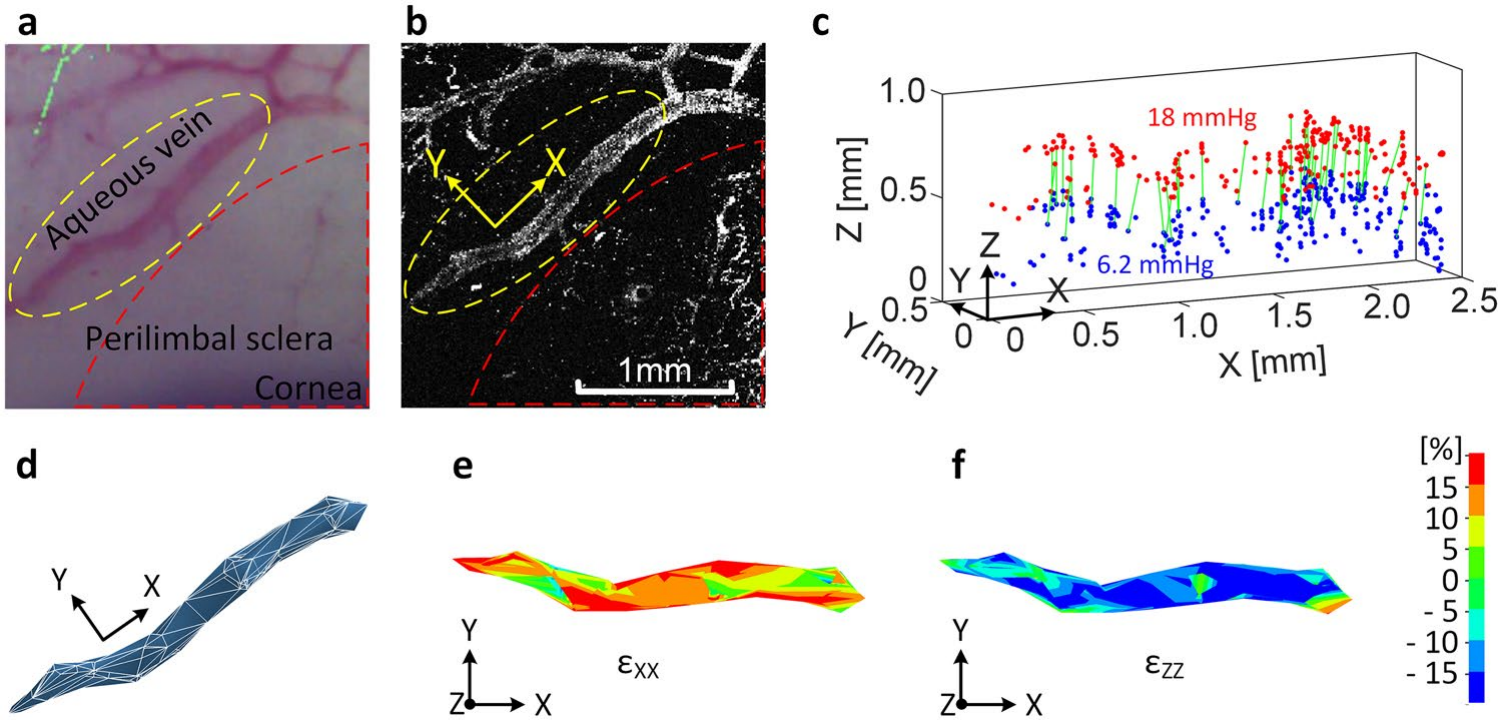
Perfused porcine globe (Example 1). (**a**) Photo of an aqueous vein and branched vein segments, (**b**) corresponding photoacoustic image, (**c**) tracked points in 3-D of the circled vein, where blue and red points are at IOPs of 6.2 and 18 mmHg, respectively, and the green displacements of select identical points showing clear outward movement as the IOP increases, (**d**) mesh with tetrahedral elements, and the spatial strain fields of (**e**) normal strain $\varepsilon$_XX_ at 18 mmHg and (**f**) $\varepsilon$_ZZ_ at 18 mmHg. In (**a**,**b**), the sclera under the red dashed line indicates a region of deeper veins. (**d**-**f**) were generated using LS-Dyna R9.0, http://www.lstc.com/products/ls-dyna.

A porcine eye was preconditioned, as described in the section of perfusion set-up and process, and perfused with the red contrast agent to stain the veins. A photo of the stained aqueous veins and a corresponding photoacoustic image are shown in Fig. 2a,b, respectively. Here, the red dashed lines delineate the area where the deeper aqueous veins are not visible in white light photograph but are resolved in photoacoustic imaging. The Z-direction is perpendicular to the scleral surface at the reference point and the X and Y axes lie roughly in the thickness of the sclera with X along the longitudinal direction of the yellow-circled vein and Y along its transverse direction. The tissue was imaged along the Z-axis. The strain at the lowest pressure (6.2 mmHg) establishes the near-zero reference strain state. At this pressure, the diameter of the vein is approximately 200 µm at its central location. Five photoacoustic images were taken as IOP was gradually increased during perfusion from 6.2 mmHg to 18 mmHg, which spans the porcine IOP from very low to moderate. Blue marker points shown in Fig. 2c were identified at 6.2 mmHg and tracked through 5 images. Red points in Fig. 2c indicate the locations of the tracked points at 18 mmHg. Note that the tracked points are on the inner vein wall within the sclera, not on the eye’s surface. Also shown on Fig. 2c are the displacement vectors (green lines) of a subset (for clarity) of the points. These vectors clearly reveal the outward movements of points on the vein wall toward the sclera’s outer surface as the eye expands in response to the increase in IOP. The locations of the points relative to their initial locations after this increase in IOP also confirm that the stained marker points were fixed to the vein’s wall during the experiment and so serve as displacement tracers for strain calculation. Figure 2d shows 695 undeformed tetrahedral elements at 6.2 mmHg, while Fig. 2e,f show the normal strain spatial fields $\varepsilon$_XX_ and $\varepsilon$_ZZ_, respectively, at 18 mmHg. The vein is seen to be stretched in the nearly longitudinal direction, X, while contracted in the Z-direction. Thus, the vein stretches along its length and contracts in the plane of its cross-section while its bulk form moves radially outward.

### Three-dimensional strains in porcine aqueous veins (Example 2)

**Figure 3:**
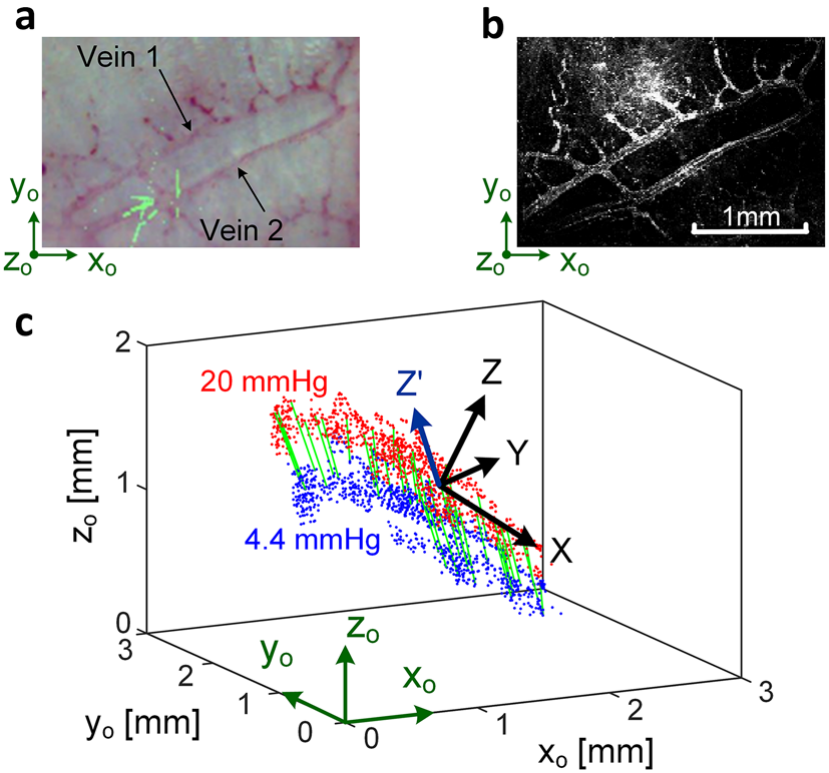
Perfused porcine globe (Example 2). (**a**) Photo of multiple aqueous veins with particular note of aqueous Vein 1 and aqueous Vein 2, (**b**) photoacoustic image, and (**c**) tracked points in 3-D, where blue and red points are at 4.4 and 20 mmHg, respectively.

**Figure 4:**
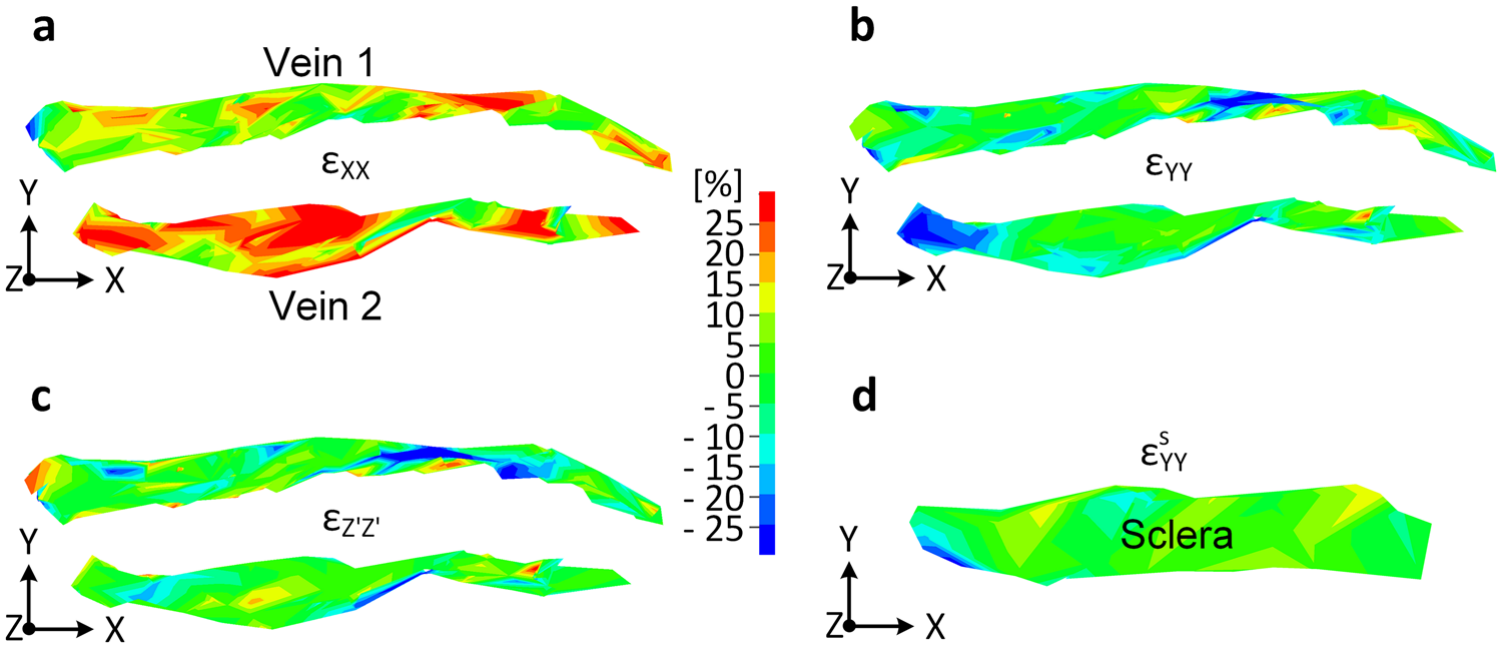
Spatial variation of normal strains at 20 mmHg. (**a**) $\varepsilon$_XX_, (**b**) $\varepsilon$_YY_, and (**c**) $\varepsilon$_Z’Z’_ of the aqueous veins 1 and 2, and (**d**) estimate of $\varepsilon$^s^_YY_ of tissue between the two aqueous veins. (Generated using LS-Dyna R9.0, http://www.lstc.com/products/ls-dyna).

Figure 3 gives results of imaging a region of perilimbal sclera of another porcine globe. A complex collection of minor vein segments branching from larger veins were revealed during perfusion as shown in the photograph Fig. 3a and the photoacoustic image Fig. 3b of the region at the reference IOP of 4.4 mmHg. Blue marker points shown in Fig. 3c were identified at the reference IOP and tracked in 3-D through 8 photoacoustic images. Red points indicate the locations of the tracked points at 20 mmHg. The spatial coordinates of the tracked points were determined using the x_o_y_o_z_o_ coordinate system where the z_o_ direction is roughly along the imaging axis of the photoacoustic microscopy system. Quantities measured in terms of the x_o_y_o_z_o_ coordinates were then transformed to the XYZ’ coordinates for the calculation of strains. Here, X and Y are oriented in the plane of the two nearly parallel veins 1 and 2, with X directed along the longitudinal direction of the veins. The Z’-axis is used rather than Z because it is perpendicular to the X-axis and its direction is nearly parallel to the direction of the displacement vectors (green lines in Fig. 3c). Figure 4a,b,c respectively show the spatial variation of the strains $\varepsilon$_XX_, $\varepsilon$_YY_, and $\varepsilon$_Z’Z’_ in the vein tissues at 20 mmHg. In Fig. 4a, the strain $\varepsilon$_XX_ is roughly directed along the length of the veins and so represents a measure of the vein’s deformation in its longitudinal direction. In Fig. 4b, the variation of the compressive strain $\varepsilon$_YY_ is shown in the veins 1 and 2 at 20 mmHg. In Fig. 4d, the strain $\varepsilon$^s^_YY_ is given in the approximately 0.35 mm wide (in the Y-direction) scleral tissue between the two veins. This was determined by the creation of a mesh across the sclera using nodal points at the vein/sclera interfaces. The computed strain from this mesh then represents the normal strain component in the scleral tissue in the Y direction. This calculation assumes the deformation of the vein walls in the Y-direction was negligible relative to the deformation of the scleral tissue between the veins.

### Variation of average strains as functions of IOP

**Figure 5:**
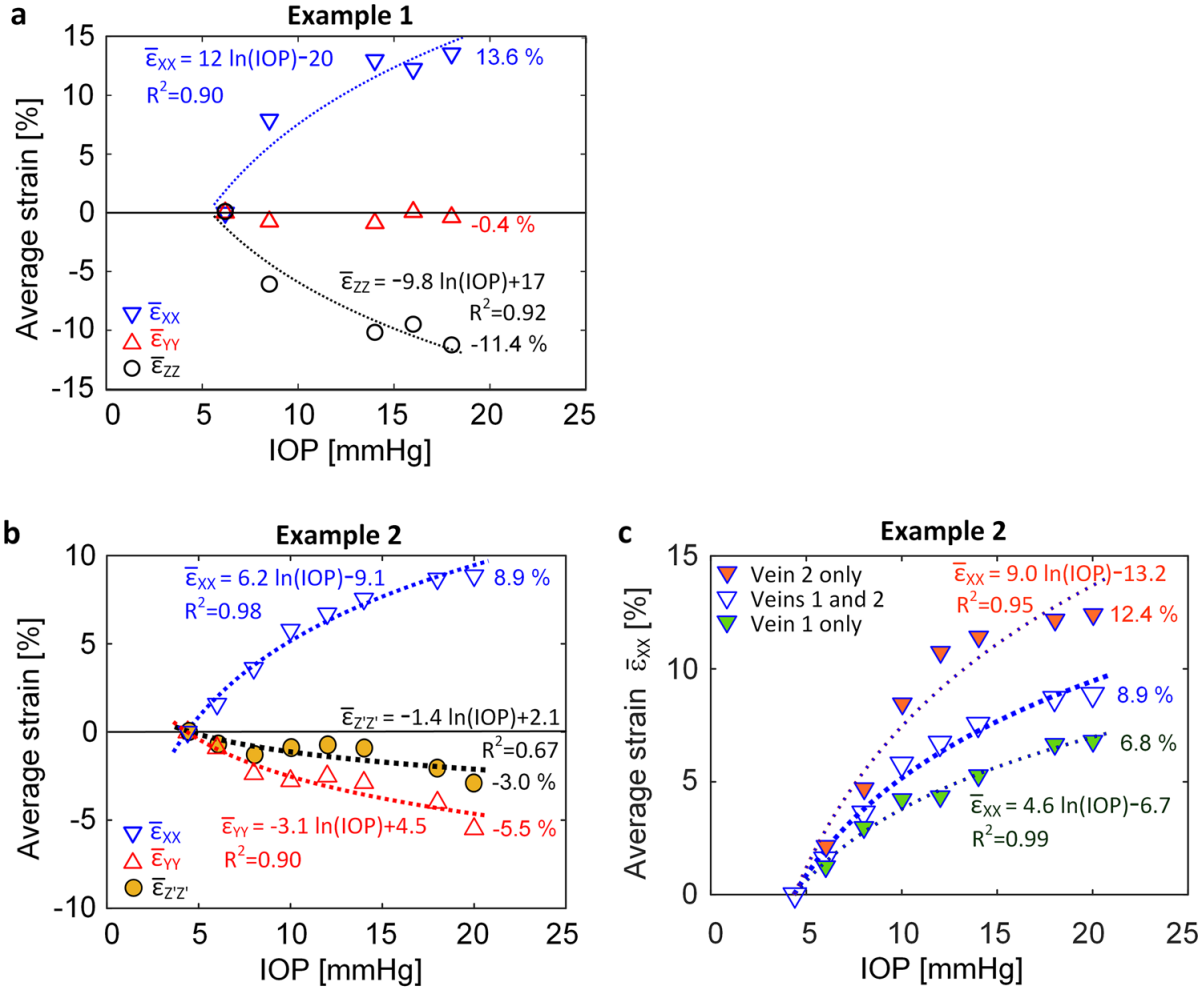
Spatial averages of the aqueous vein strain components, weighted by element volume, as functions of IOP. (**a**) Example 1 (Fig. 2) and (**b**)–(**c**) Example 2 (Figs. 3 and 4). In (**b**), the averages were computed over the combined regions of aqueous veins 1 and 2. In (**c**), $\overline{\upvarepsilon }$_XX_ was determined separately over the regions of vein 1 and vein 2, as well as over the combined regions of aqueous veins 1 and 2. Also shown in (**a**)–(**c**) are dashed line logarithmic fits to the data and the coefficient of determination, R^2^.

Figures 5a-c show $\overline{\upvarepsilon }$_XX_, $\overline{\upvarepsilon }$_YY_, $\overline{\upvarepsilon }$_ZZ_, and $\overline{\upvarepsilon }$_Z’Z’_, the volume-weighted average normal strains in the X-, Y-, Z-, and Z’-directions, respectively, as functions of IOP. Figure 5a was obtained from aqueous vein Example 1 (Fig. 2), whereas Fig. 5b,c are from Example 2 (Figs. 3 and 4). In Fig. 5b, the averages for each strain component were computed over the region spanned by both veins 1 and 2 together. In Fig. 5c the average strain $\overline{\upvarepsilon }$_XX_ was computed separately over the regions of vein 1 and vein 2. As most soft tissues stiffen with increases of strain, logarithmic functions were fit to the measured strains as functions of IOP in Fig. 5a–c. The figures show the dotted line curve fits, their functions and goodness fit values, R^2^. In Fig. 5a–c, the longitudinal strain $\overline{\upvarepsilon }$_XX_ in both examples is seen to be almost entirely tensile along the veins’ lengths and to increase logarithmically with IOP in both experimental cases. In addition to the longitudinal strain component, logarithmic variation with IOP was also found to occur, with reasonable goodness of fits, for the transverse strain components $\overline{\upvarepsilon }$_ZZ_ in Example 1 (Fig. 5a) and $\overline{\upvarepsilon }$_YY_ in Example 2 (Fig. 5b).

In Example 1, the vein’s normal strain in the Y-direction was directed in the plane of the scleral wall and $\overline{\upvarepsilon }$_YY_ was found to be slightly compressive as shown in Fig. 5a. The magnitude of this strain component is the result of substantial lateral compression due to a Poisson contraction induced by the vein stretch in the X-direction, combined with its interaction with the sclera which is in tension in the Y-direction. The resulting very small strain over this range of IOP makes this strain component a poor fit to an exponential function.

In Example 2 (Fig. 5b), the variation of $\overline{\upvarepsilon }$_Z’Z’_ with IOP is poorly fit by the exponential function (R^2^ = 0.67) possibly because it is not directed radially like $\overline{\upvarepsilon }$_ZZ_ in Example 1 (Fig. 5a), so its relationship to IOP is more complex because it is not normal to the sclera’s thickness. In Fig. 2f (Example 1), $\varepsilon$_ZZ_ is mainly compressive which would be expected given the compression of the sclera in the globe’s wall-thickness (i.e. radial) direction as the globe expands. Furthermore, the average strain component $\overline{\upvarepsilon }$_ZZ_ is found in Fig. 5a to be significantly exponentially related to IOP, with R^2^ = 0.92.

## Discussion

This manuscript describes a method for 3-D displacement imaging and computation of strain in the aqueous veins residing in the perilimbal sclera of eyes. The method is demonstrated by two representative example experiments using ex vivo, porcine, globes. To the authors’ knowledge, these are the only such quantitative, data driven, calculations of the aqueous vein strains available in the open literature. Also, this method is applicable to imaging and strain analysis of other tissues provided contrast agent can be delivered or endogenous features can be discerned. In the whole globe experiments, a perfused contrast agent was used to establish trackable feature points on the inner surface of the vein walls. Imaging was conducted using photoacoustic microscopy measurements of vein spatial features during perfusion. Strain computations were made using the strain–displacement engine of a finite element package, based on a deforming mesh of the tracked points as the veins deformed due to IOP and outflow. Results based on the methods were validated against two accepted standard measurement methods for surface strain cases, as well as another publication. Results from whole globe perfusion experiments revealed complex strain fields in the veins dependent on IOP as well as vein anatomy and position in the sclera. Veins were observed to move radially outward during increases in IOP and to possess significant spatial strain variation, possibly influenced by their arrangement and branching patterns.

The photoacoustic microscopy system in this study successfully resolved the spatial features in the validation experiments in Supplementary Figs. S1 and S3. The system also resolved the aqueous veins perfused with an optical contrast agent in 3-D, as shown in Figs. 2 and 3. As marked by the red dashed contours in the images in Fig. 2a,b, the system was able to visualize aqueous veins within the sclera in the photoacoustic microscopy image Fig. 2b that were not visible in white light microscopy Fig. 2a. In addition, by targeting the specific optical absorption profile of the contrast agent, photoacoustic microscopy produced very high contrast over the irrelevant tissue components as can be seen in Figs. 2b and 3b. These photoacoustic images also show that photoacoustic microscopy provided relatively uniform imaging sensitivity to vessels with varied diameters. Flow-based imaging technologies such as optical coherence angiography, depending on the thresholding of decorrelation values, have shown non-uniform sensitivities to vessels with varied dimensions and flow rate^30^. In contrast, photoacoustic microscopy can reliably capture optical contrast even if the optical absorbers are static. All of these considerations make photoacoustic microscopy suitable for spatial feature tracking and observing the biomechanical behaviors of aqueous veins in ex vivo globes.

A dynamic finite element software package was used for the strain calculations. Given the input of a series of deforming meshes, only the strain–displacement portion of the software was used; material properties were irrelevant since stresses were not studied. The approach in the present manuscript requires the creation of a finite element mesh from the reference image, and the development of an FEA model. However, the resulting model can readily provide further mechano-biological context for the measured behavior through subsequent calculations of stresses using the same model and a suitable constitutive formulation among those available in the program (given the tissue properties). Additionally, the model can be used in an inverse method^31,32^, with a connected package such as LS-OPT (Livermore Software Technology, Livermore, CA), to determine the properties based on a constitutive formulation. Additionally, the FEA system can conduct immediate tensor transformations to determine strains in any directions of interest, such as the principal directions that indicate the extrema of the strains, or in any coordinate system, including curvilinear.

A concern during the development of the methods was the necessity that the contrast agent establish contrast features attached to the vein. Thus, the images Figs. 2c and 3c were critical to show that the tracked points were indeed fixed to the inner vein wall and not flowing in the vein during perfusion. Thus, the measured displacement of a point represents the movement of that physical point of the vein wall. The finite element mesh is formed from a group of such points. Strains are calculated from the formulas representing the change in shape of the mesh as the points displace and so are (nondimensional) measures of the deformations of the tissue to which the points are attached. In most cases, the mesh is formed from a group of points on the vein wall. In Fig. 4d of Example 2, a mesh is formed across the sclera using points on the veins that border the sclera.

In the Supplementary Information file, the accuracy of the measurement technique was assessed by inducing known displacements in the photoacoustic reference image for the experiment in Supplementary Fig. S1a. When the numerically induced strain was 9.53% in the direction of stretch, the average strain determined from all elements by the photoacoustic microscopy-FEA technique was 9.56%, resulting in a 0.03% strain difference and 0.31% error. The mean (0.16 × 10^–3^), standard deviation (5.0 × 10^–3^), and average absolute error (3.7 × 10^–3^) were also determined from the differences between the measured elemental strains and the induced strain of 9.53%. These values are similar to those calculated for a study of posterior sclera^20^ when the difference in input strains are considered (2% input strain^20^ versus 9.53% in present manuscript). These error values are sufficiently small to not introduce appreciable error into the calculations of strain in the present manuscript.

The logarithmic dependence of strain on IOP found in Fig. 5a–c is consistent with tissue stiffening commonly observed in specimen tensile testing.

In Example 2 the imaged region was a tight circuit of veins around a region of sclera, and included multiple branches. In Fig. 5c, the average strain $\overline{\upvarepsilon }$_XX_ of vein 2 (12.4%) at 20 mmHg was substantially higher than that of vein 1 (6.8%). It is conjectured that the multiple branches of vein 1 (see Fig. 3a, b) have an effect of lowering vein pressure in that branch, and hence the longitudinal strain in that vein’s wall.

It should be noted that understanding of the vein response is complicated by their orientation in the wall of the sclera. Due to the orientation of the veins in the sclera in Example 2, the Z-direction is perpendicular to the XY plane of the two veins, but it is not the radial direction of the globe. In Fig. 3c, a coordinate transformation about the X-axis was made to produce the Z’-axis that is nearly along the direction of the outward movements of tracked points and the Y’Z’ plane is roughly parallel to the cross-section of the veins. (For clarity, the Y’ axis is not shown on Fig. 3c).

Since the methods described in this study target calculation of the dynamic strain of the aqueous veins during the variation of IOP, rapid image acquisition is desired. The imaging speed of the present system is limited by a 5 kHz laser repetition rate. A laser with a higher repetition rate will significantly increase the data acquisition rate reducing changes of strain during the acquisition. Also, the photoacoustic microscopy system in this study has axial resolution that is limited by the receiving bandwidth and aperture of the ultrasound detector, i.e. the needle hydrophone. Since scleral tissue has limited thickness^33^, using ultrasound transducers with higher receiving frequency and larger aperture may further improve the axial resolution without sacrificing the capability of observing the deeply embedded aqueous veins.

This technique does not quantify or measure the flow within the vein. Strains in the veins and the immediate sclera are the result of three factors, IOP, venous pressure and flow stresses. The relative, quantitative contribution of these factors to the strains is difficult to parse and would require additional study design having that specific objective.

## Conclusions

This manuscript demonstrates a method for 3-D imaging using a perfused contrast agent and finite element modeling to determine strains within complex biological tissue containing sclera and veins. The method was validated against simpler surface strain experiments. Two experiments using porcine globes gave strain fields in the aqueous vein tissues as a function of IOP. The veins simultaneously deformed and moved radially outward as IOP was increased, which is logical given that the increase in volume from perfusion induces expansion of the globe. This method will be advanced for additional studies to determine the interplay between tissue biomechanics and the aqueous veins in order to understand IOP regulation and aqueous humor outflow. We anticipate that this knowledge could lead to testing if tissue biomechanics and aqueous veins before surgery correlate with Schlemm’s canal-based surgery outcomes.

## Supplementary Information

Supplementary Information.

## Data Availability

The data analyzed in this article are included in the main text, provided in the Supplementary Information file, or available from the corresponding author on reasonable request.
