# Supplementary material for: Method for the biomechanical analysis of aqueous veins and perilimbal sclera by three-dimensional photoacoustic imaging and strain field calculation
Source: Sci Rep. 2021 Nov 11;11:22108. doi: 10.1038/s41598-021-01458-1 (PMC8585983; doi:10.1038/s41598-021-01458-1)
Supplement: Supplementary file 1 — Supplementary Information. [file 41598_2021_1458_MOESM1_ESM.pdf]

Method for the biomechanical analysis of aqueous veins and perilimbal sclera by three-dimensional photoacoustic imaging and strain field calculation

Supplementary Information

Linyu Ni[1] nilinyu@umich.edu
John Riesterer[2] riestere@umich.edu
Huaizhou Wang[3,4] trhz_wang@163.com
Layla Berry[2] lhberry@umich.edu
Kara Blackburn[3] kablackb@umich.edu
Jonathan Chuang[3] jochuang@umich.edu
Wonsuk Kim[2] wskim@umich.edu
Guan Xu[1,3] guanx@med.umich.edu
Sayoko E. Moroi[3,5] sayoko.moroi@osumc.edu
Alan Argento[2,*] aargento@umich.edu

[1]Department of Biomedical Engineering
University of Michigan, Ann Arbor, MI 48109, USA

[2]Department of Mechanical Engineering
University of Michigan-Dearborn
4901 Evergreen Road, Dearborn, MI 48128, USA

[3]Department of Ophthalmology and Visual Sciences, Kellogg Eye Center
University of Michigan, Ann Arbor, MI 48105, USA

[4]Currently at Beijing Tongren Eye Center, Beijing Tongren Hospital
Capital Medical University, Beijing, China

[5]Department of Ophthalmology and Visual Sciences, Havener Eye Institute
The Ohio State University Wexner Medical Center, Columbus, OH 43210, USA

*Corresponding author:
Tel: +1-313-593-5029
Fax: +1-313-593-3851
E-mail: aargento@umich.edu

Mechanical and numerical validations

The photoacoustic microscopy-FEA method is validated in the Supplementary Information file by: (1) comparison of average strains in a low modulus elastomer determined by a conventional tensile test machine to those simultaneously measured by photoacoustic microscopy-FEA; (2) comparison of 3-D surface strains on the posterior region of a whole porcine globe determined by photoacoustic microscopy-FEA to those measured by a widely-used commercial strain imaging system and another published strain field for the posterior of a whole bovine eye. In addition, the accuracy of feature tracking and strain calculation is numerically evaluated for Case (1) using a recently used[1] error calculation method.

Tensile test of an elastomer specimen. A small, custom-made, tensile test apparatus placed under the lens of the photoacoustic microscopy system was used to apply uniaxial tensile loading to a specimen during simultaneous photoacoustic microscopy-FEA imaging. The tensile test apparatus consists of a pair of in-line grips attached to a pair of micrometers that are fixed to mounting structures at the free ends of the tensile specimen. Comparison was made between average strains in the specimen determined by the micrometer readings and by photoacoustic microscopy-FEA. To avoid tissue specimen hydration issues, the validation test was conducted on a low modulus, thermoplastic elastomer tensile test specimen. The rectangular elastomer specimen was extracted from a larger sheet of 1 mm thick using a straight razor. A pattern of speckles created on the specimen by lightly sprayed red paint was used to create contrast tracking points. After mounting in the grips, the resulting test specimen dimensions were approximately 13 mm x 3.1 mm x 1 mm (length x width x thickness). The specimen was deformed in 0.05 mm increments by adjustment of the micrometers while photoacoustic imaging, tracking and strain calculation were conducted after each step using the methods described in the main article. The average normal strain in the X-direction, $\bar{\varepsilon}_{XX}$, in the direction of loading determined by the tensile test machine was calculated using the one dimensional true strain formula, $\bar{\varepsilon}_{XX} = \ln(1+ \Delta L/L)$, where ΔL is the specimen's change in length given by the micrometer readings, and L is its original length. Comparison was made between this value and the average true strain determined by the photoacoustic microscopy system. Additionally, Poisson's ratio, ν, was estimated from the photoacoustic microscopy-FEA measured values using $\nu = -\bar{\varepsilon}_{YY}/\bar{\varepsilon}_{XX}$ where $\bar{\varepsilon}_{YY}$ is the average strain in a transverse direction perpendicular to the axis of tensile loading.

Accuracy of feature tracking and strain calculation. In order to quantify the accuracy of the point tracking and strain calculation, rigid body translations and uniform deformations were numerically induced in the reference image[1]. Firstly, 0.3 mm translation was applied in the direction of stretching followed by 0.2 mm translation in the perpendicular direction. Secondly, tensile strain of approximately 10% was generated uniformly in the stretching direction of the translated image, and then compressive strain of approximately 5% was uniformly induced in the direction perpendicular to stretching. Contrast points were tracked and strains were calculated between the reference and numerically modified images. Finally, bias, uncertainty and average absolute error were evaluated.

Whole porcine globe surface imaging. Three-dimensional strains on the posterior surface of whole porcine globes undergoing perfusion were measured using photoacoustic microscopy-FEA and a commercial strain imaging system (ARAMIS 3-D, GOM GmbH, Braunschweig, Germany)

equipped with a pair of digital cameras and software for image correlation. The imaged region was a portion of the optic nerve head (ONH) and the immediate surrounding sclera. This region was selected because the abrupt change in anatomy at the interface between the optic nerve head and the sclera produce large gradients of strain that are challenging to a strain imaging system. As in the tensile validation test, paint speckles were applied to the surface of the eyes to serve as contrast tracking points. In both experiments in this section, strains were determined as the eye's IOP was varied by perfusion with BSS using a syringe pump. In the photoacoustic microscopy-FEA experiment, points were imaged and a mesh generated, followed by strain calculations using FEA, as described in the main article. In the experiment using the commercial software, the system automatically identified feature points to track and compute the 3-D strains. In both cases, strains are presented relative to an arbitrary reference state (10 mmHg). Quantitative and qualitative comparisons of the 3-D strain fields measured by the two systems were made.

Validation results

Tensile test: mechanical validation. Fig. S1a shows a portion of the elastomer specimen mounted in grips with an overlaid grayscale photoacoustic image and mesh. Only the left side of the symmetric specimen was imaged and the overall dimension of the undeformed mesh with 52 triangular elements is approximately 3.56 mm by 2.56 mm as shown in the figure. The X and Y axes indicate the longitudinal and transverse directions, respectively, of the specimen. The specimen was stretched in the X-direction. Fig. S1b shows a photoacoustic image with overlaid points identified and tracked from the undeformed reference image and two deformed images when the grip separation distance was increased by 0.55 and 1.4 mm. Fig. S1c shows the deformed mesh and the photoacoustic microscopy-FEA color map of the true normal strain in the X-direction, ε_{XX}, at the end of the 1.4 mm stretching process. Local strain concentrations can be seen in the strain map at the left end of the image due to clamping stress at the grip. These strains diminish toward the center of the specimen, as seen in the image. The same effect occurs at the non-imaged right grip. Stress and strain non-uniformities near grip locations are typical in mechanical testing, and are not captured by the average values given by the test machine measurements.

Fig. S2a shows the average of the strain component ε_{XX} in the imaged region determined from photoacoustic microscopy-FEA versus the strain measurement determined by the test machine using the distance between the two grips. The measured average elemental strain $\bar{\varepsilon}_{XX}$ of the imaged region is about 9.4% at the end of the test and the corresponding test machine measured strain is 10.4%. The 1% strain difference between the two measurements is reasonable considering the strain non-uniformities captured throughout the specimen by photoacoustic microscopy-FEA that are not captured by the test machine measurements. The relationship between the photoacoustic microscopy-FEA measurements and the test machine measurements is seen to be linear with the coefficient of determination (R^2) value of 0.995. The high correlation indicates the validity of the photoacoustic microscopy-FEA strain measurements at all the levels of tested strains. Fig. S2b shows the relationship between the photoacoustic microscopy-FEA averages of the transverse strain ε_{YY} and ε_{XX}. The slope of the linear curve-fit line is 0.53 which gives a good approximation of the elastomer material's Poisson's ratio which is known to be approximately 0.5.

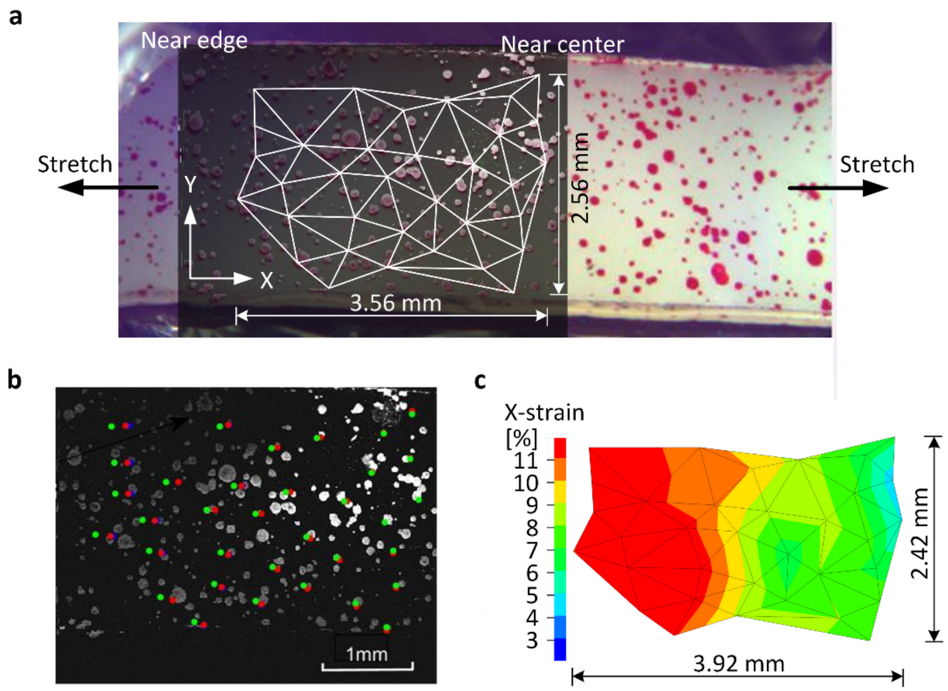

Figure S1. Tensile test validation of an elastomer specimen. (a) Photo of an undeformed elastomer specimen with an overlaid grayscale photoacoustic image and mesh, (b) a photoacoustic image with overlaid points identified and tracked from the reference image (blue dots) and two deformed images stretched 0.55 mm (red dots) and 1.4 mm (green dots), and (c) the deformed mesh with the color map of the strain ε_{XX} at the end of the stretch test, generated using LS-Dyna R9.0, http://www.lstc.com/products/ls-dyna.

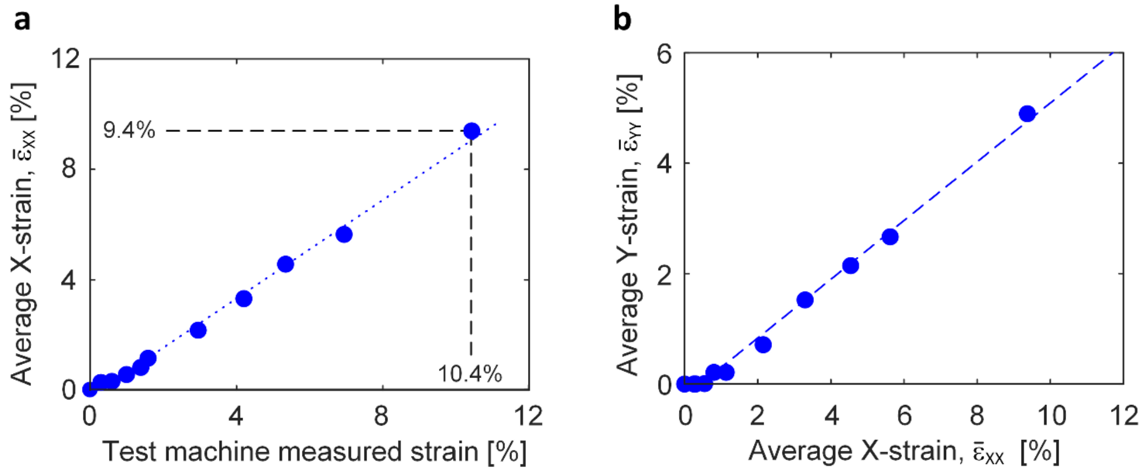

Figure S2. Average strains of an elastomer specimen. (a) Average strain $\bar{\varepsilon}_{XX}$ determined by photoacoustic microscopy-FEA versus strain determined from the distance between the test machine grips, (b) average strain $|\bar{\varepsilon}_{YY}|$ (absolute value of compressive strain in the Y-direction) versus $\bar{\varepsilon}_{XX}$ determined by photoacoustic microscopy-FEA.

Tensile test: numerical validation. Using the method described in [1] both rigid body translations and uniform strains were numerically induced in the photoacoustic image of Fig. S1a, followed by feature tracking of the image points and strain calculation. When the numerically induced strain is 9.53% in the X-direction, the average strain $\bar{\varepsilon}_{XX}$ determined from all elements by the photoacoustic microscopy-FEA technique is 9.56%, resulting in a 0.03% strain difference and 0.31% error. The strain error of each element is also determined as the difference between the numerically induced strain of 9.53% and the elemental ε_{XX}. From the strain errors of all elements the mean (bias) and standard deviation (uncertainty) are calculated. In addition, the mean of the absolute values of the strain errors (average absolute error) is determined[1]. Calculations of bias, uncertainty and average absolute error respectively are 0.16×10^{-3} (that is, 0.016% strain), 5.0×10^{-3} (that is, 0.5% strain), and 3.7×10^{-3} (that is, 0.37% strain).

Whole porcine globe surface strain. Results for the posterior surface strains of a porcine globe are given in Figs. S3-S4. Points in the gray shaded region in Fig. S3a were tracked in 3-D as IOP was increased from 10 mmHg to 21 mmHg during perfusion. The deformed mesh and photoacoustic microscopy-FEA strain ε_{YY} at the IOP of 21 mmHg are shown in Figs. S3b and S3c in two different views. The tracked points do not lie in the XY-plane, as seen in Fig. S3c. The averages of ε_{XX} and ε_{YY} over the entire imaged region are 1.04% and 1.03%, respectively. The strains ε_{XX} and ε_{YY} of the element *C* in the ONH region of Fig. S3b are 1.9% and 1.5%, respectively, which are substantially larger than the overall average strains.

For comparison, posterior strains were obtained from another test on a porcine globe using the commercial strain imaging system. Normal strains ε_{XX} and ε_{YY} at the IOP of 21 mmHg are given in Figs. S4a and S4b, respectively. At the central location of the ONH, point C in Fig. S4, ε_{XX} and ε_{YY} are approximately 1.6 and 1.3%, respectively. These values are in reasonable agreement with those of the element *C* in Fig. S3b considering Figs. S3 and S4 are from different tests on different eyes. Additionally, the strains in the boundary region around the ONH are approximately 1% (yellow color) in both Figs. S3 and S4 which further confirms the similarity of the measurements made by the two different imaging systems. It should also be noted that in order to achieve sufficient tracking in the case of the commercial imaging system, more paint was needed than in the case of the photoacoustic microscopy system. Surface paint tends to stiffen the tissue, thereby lowering surface strains, as seen in the case of the commercial imaging system.

The validation experiments in Figs. S3 and S4 were challenging because of known abrupt changes in geometry and stiffness at the junction between the nerve and the sclera that induce large strain gradients. Relatively high strain in the region of the ONH, and its sharp decrease across the interface with the sclera were similarly captured by the commercial system and the photoacoustic microscopy-FEA system. A similar trend was observed for the optic nerve head of the bovine eye[2] based on measured displacement magnitudes (strains were not calculated).

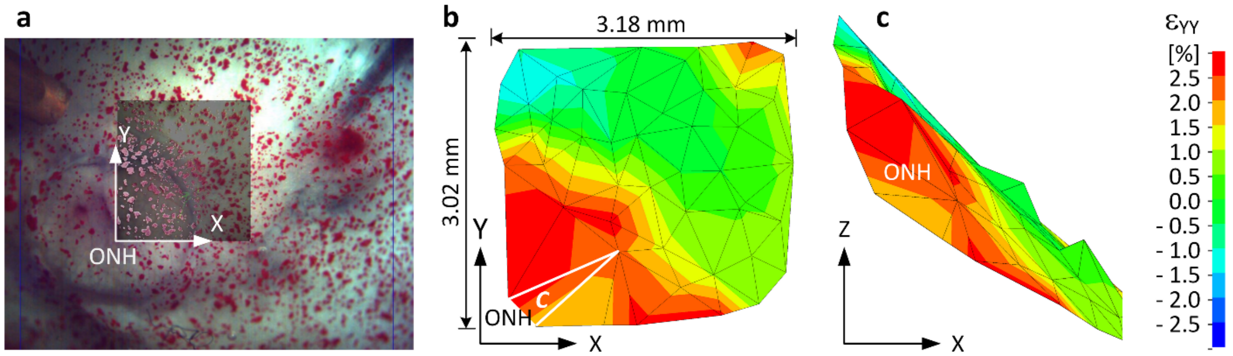

Figure S3. Whole globe surface imaging. (a) Porcine globe with sprayed speckles. Photoacoustic image in grayscale is overlaid on the photo. (b) Deformed mesh of 78 elements showing the spatial variation of ε_{YY} at 21 mmHg in the XY-plane view, and (c) ε_{YY} at 21 mmHg in the XZ-plane view. (b) and (c) were generated using LS-Dyna R9.0, http://www.lstc.com/products/ls-dyna.

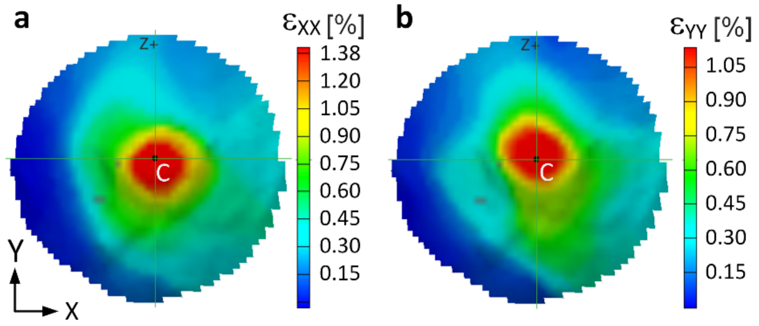

Figure S4. Surface strains measured by a commercial imaging system. (a) ε_{XX} and (b) ε_{YY} in the ONH region of a porcine globe at 21 mmHg, generated using ARAMIS 3D v.5.4.1, http://www.gom.com/en/products/3d-testing/aramis-3d-camera.

References

1 Midgett, D. E., Jefferys, J. L., Quigley, H. A. & Nguyen, T. D. The inflation response of the human lamina cribrosa and sclera: Analysis of deformation and interaction. *Acta Biomaterialia* **106**, 225-241 (2020).

2 Myers, K. M., Coudrillier, B., Boyce, B. L. & Nguyen, T. D. The inflation response of the posterior bovine sclera. *Acta Biomaterialia* **6**, 4327-4335 (2010).
